# Supplementary material for: Facilitating bootstrapped and rarefaction-based microbiome diversity analysis with q2-boots
Source: F1000Res. 2025 Jan 15;14:87. [Version 1] doi: 10.12688/f1000research.156295.1 (PMC12489448; doi:10.12688/f1000research.156295.1)
Supplement: Peer review history

# Reviewer report: Reviewer response for version 1

**DOI:** 10.5256/f1000research.171585.r374737

**Published:** 2025-05-05

**Authors:** Luke R Thompson

**Affiliations:** 1 Northern Gulf Institute, Mississippi State University, Oktibbeha, USA

This articles describes a new QIIME 2 plugin, q2-boots, which provides bootstrapping and rarefaction of feature tables, perform alpha and beta diversity analysis on those tables, and summarize the output. The article provides an overview of the plugin and its commands, plus an example dataset and analysis. Much more detailed guidance and a full tutorial are provided in the online documentation. This software should be broadly useful to microbiome researchers and represents a valuable contribution to the microbiome field.

 Abstract

 - In the first sentence of the results section, it might be helpful to insert "n times—with replacement (bootstrapped) or without replacement (rarefied)—and compute" to define these two related terms in the abstract.

 - In the conclusion, it's unclear why the fact that the three metrics for averaging beta-diversity matrices being highly correlated would be relevant. Could you briefly add that this allows selection of a more computationally efficient metric with minimal tradeoffs?

 Keywords

 - Suggest adding "environmental DNA", "metabarcoding", and "amplicon sequencing", as these are related fields/techniques that share many features with microbiome research and whose practitioners may be interested in this plugin.

 Background

 - In second sentence, suggest changing "microbiome researchers and data scientists" to "microbiome and environmental DNA researchers and other data scientists working with biological feature tables."

 - I agree with the other reviewer that some additional background on why bootstrapping/rarefaction are important would be helpful. Just a few sentences summarizing the two papers by Schloss would provide more justification for using this plugin.

 - Related, the advantages to researchers of using this plugin could be highlighted in the figures and perhaps in the Conclusions.

 Figures

 - The fonts in the workflow diagrams are very small. Would it be possible to make them bigger, perhaps by adding line breaks?

 - The figures do not help show what the tool does or why it's useful. Would it be possible to include some figures showing how bootstrapping/rarefaction improve an analysis? These could be extracted from the tutorial. A short explanation of the value added by this plugin could go in the main text, perhaps in the Conclusions.

Are the conclusions about the tool and its performance adequately supported by the findings presented in the article?

Yes

Is the rationale for developing the new software tool clearly explained?

Yes

Is the description of the software tool technically sound?

Yes

Are sufficient details of the code, methods and analysis (if applicable) provided to allow replication of the software development and its use by others?

Yes

Is sufficient information provided to allow interpretation of the expected output datasets and any results generated using the tool?

Yes

Reviewer Expertise:

Microbiome and environmental DNA research, including metabarcoding analysis and data science

I confirm that I have read this submission and believe that I have an appropriate level of expertise to confirm that it is of an acceptable scientific standard, however I have significant reservations, as outlined above.

# Reviewer report: Reviewer response for version 1

**DOI:** 10.5256/f1000research.171585.r374740

**Published:** 2025-04-18

**Authors:** Vitor Heidrich

**Affiliations:** 1 CIBIO, Universita degli Studi di Trento, Trento, Trentino-Alto Adige/South Tyrol, Italy

The authors developed a QIIME 2 plugin allowing to perform diversity analyses following repeated rarefaction of feature-tables followed by summarizing the results generated by each rarefaction run. I agree with the authors that the plugin developed was much needed by the QIIME 2 community, as I also have followed the discussion brought to light by PD Schloss in his recent papers and was surprised to realize it was not possible to perform repeated rarefaction in QIIME 2. The rational for the plugin is clearly and concisely stated, as well as the actions implemented in it. The authors were also very fair in stating that other normalization techniques (possibly more tailored to a particular kind of analysis) do exist. Although not essential for the understanding of this work, I only wonder whether mentioning some examples of those other non-rarefaction-based normalization approaches (or specifically those available in QIIME 2) could be useful for the reader (could be an "e.g." in this sentence of the Conclusions: "or as a basis for comparison against more sophisticated normalization techniques").

Are the conclusions about the tool and its performance adequately supported by the findings presented in the article?

Yes

Is the rationale for developing the new software tool clearly explained?

Yes

Is the description of the software tool technically sound?

Yes

Are sufficient details of the code, methods and analysis (if applicable) provided to allow replication of the software development and its use by others?

Yes

Is sufficient information provided to allow interpretation of the expected output datasets and any results generated using the tool?

Yes

Reviewer Expertise:

Computational biology, metagenomics, human microbiome

I confirm that I have read this submission and believe that I have an appropriate level of expertise to confirm that it is of an acceptable scientific standard.

# Reviewer report: Reviewer response for version 1

**DOI:** 10.5256/f1000research.171585.r370290

**Published:** 2025-04-10

**Authors:** Digvijay Verma

**Affiliations:** 1 Babasaheb Bhimrao Ambedkar University, Lucknow, India

The manuscript introduces q2-boots, a plugin for QIIME 2 aimed at simplifying the analysis of microbiome diversity through bootstrapping and rarefaction techniques. The authors present a comprehensive set of functionalities that allow for the calculation of thirty distinct alpha diversity metrics and twenty-two beta diversity metrics on resampled or rarefied feature tables. This tool's incorporation into the QIIME 2 framework is a significant advancement in microbiome research, effectively filling a notable gap in existing QIIME 2 methodologies. The manuscript clearly outlines the various functionalities offered by q2-boots and their integration within the broader QIIME 2 ecosystem. Furthermore, the authors provide a thorough comparison of bootstrapping and rarefaction-based diversity analyses, demonstrating a strong correlation between the results derived from both approaches. This analysis addresses a critical methodological issue and reassures users regarding the effectiveness of either method. In summary, this manuscript makes a significant contribution to the QIIME 2 ecosystem and the field of microbiome research as a whole. The user-friendly integration of bootstrapping and rarefaction-based diversity analysis has the potential to improve the rigor and reproducibility of microbiome studies. The writing is clear and showcases robust experimental validation, making this tool a valuable addition to the QIIME 2 suite.

 With minor clarifications and further details on certain methodological aspects, it could serve as an even more beneficial resource for the researchers. The following suggestions may be incorporated to further improve the manuscript.

 While the authors describe three methods for averaging distance matrices—medoid, non-metric-median, and non-metric-mean—there could be more discussion on the implications of each method for downstream analysis. Specifically, the manuscript mentions that all methods provide highly similar results in the specific case study but does not delve deeply into which approach might be preferable in various contexts (e.g., for different types of microbiome data or diversity metrics). Additional guidance on when to use each method would be valuable for users.

 Although the manuscript mentions the parallel computing capabilities, a more detailed discussion on the performance of q2-boots with large datasets, for example, when n > 100, would be beneficial. The results from the HEC experiment are promising, but the paper could emphasize how q2-boots scales with dataset size and the corresponding computational requirements e.g., memory usage.

 The manuscript does not mention how q2-boots interacts with other QIIME 2 plugins outside of the core-diversity workflows. For example, it would be helpful to discuss any potential compatibility with statistical or visualization tools in QIIME 2, such as q2-longitudinal or q2-phylogeny. This would give users a better idea of how q2-boots fits within a larger analysis pipeline.

 A more detailed example of how q2-boots can be used in practice, including a step-by-step guide or case study, would help potential users understand how to incorporate it into their workflows. This could be particularly useful for new QIIME 2 users.

 A more explicit discussion on the limitations of bootstrapping and rarefaction in microbiome studies, and how q2-boots addresses or mitigates these limitations, would be helpful. The authors briefly mention the controversy around rarefaction but could benefit from elaborating on the pros and cons of rarefaction versus bootstrapping and under what conditions each method might be preferred.

**Minor  suggestions:**

 Please double-check for consistency in the use of terms, particularly the distinction between "alpha diversity" and "beta diversity."

 Clarify the averaging method for diversity vectors (e.g., median by default) in relevant sections for better transparency.

Are the conclusions about the tool and its performance adequately supported by the findings presented in the article?

Yes

Is the rationale for developing the new software tool clearly explained?

Yes

Is the description of the software tool technically sound?

Yes

Are sufficient details of the code, methods and analysis (if applicable) provided to allow replication of the software development and its use by others?

Yes

Is sufficient information provided to allow interpretation of the expected output datasets and any results generated using the tool?

Yes

Reviewer Expertise:

Metagenomics, Microbial divrsity, Extremozymes

I confirm that I have read this submission and believe that I have an appropriate level of expertise to confirm that it is of an acceptable scientific standard, however I have significant reservations, as outlined above.
